# NMR-Based Multi Parametric Quality Control of Fruit Juices: SGF Profiling

**DOI:** 10.3390/nu1020148

**Published:** 2009-11-18

**Authors:** Manfred Spraul, Birk Schütz, Peter Rinke, Susanne Koswig, Eberhard Humpfer, Hartmut Schäfer, Monika Mörtter, Fang Fang, Ute C. Marx, Anna Minoja

**Affiliations:** 1 Bruker BioSpin GmbH, 76287 Rheinstetten, Germany; Email: birk.schuetz@bruker-biospin.de (B.S.); eberhard.humpfer@bruker-biospin.de (E.H.); hartmut.schaefer@bruker-biospin.de (H.S.); monika.moertter@bruker-biospin.de (M.M.); fang.fang@bruker-biospin.de (F.F.); ute.marx@bruker-biospin.de (U.M.); 2 SGF International e.V., 55286 Nieder-Olm, Germany; Email: rinke@sgf.org (P.R.); koswig@sgf.org (S.K.); 3 Bruker BioSpin S.r.l., 20133 Milan, Italy; Email: anna.minoja@bruker.it

**Keywords:** nuclear magnetic resonance spectroscopy, non-targeted screening, quantification

## Abstract

With SGF Profiling™ we introduce an NMR-based screening method for the quality control of fruit juices. This method has been developed in a joint effort by Bruker BioSpin GmbH and SGF International e.V. The system is fully automated with respect to sample transfer, measurement, data analysis and reporting and is set up on an Avance 400 MHz flow-injection NMR spectrometer. For each fruit juice a multitude of parameters related to quality and authenticity are evaluated simultaneously from a single data set acquired within a few minutes. This multimarker/multi-aspect NMR screening approach features low cost-per-sample and is highly competitive with conventional and targeted fruit juice quality control methods.

## 1. Introduction

Quality control is a constant challenge in food and life science industry with respect to contaminations and frauds like wrong labeling of the product type or the type and origin of ingredients. NMR spectroscopy combined with statistical multi parametric analysis techniques, is an extremely versatile tool to address these challenges. One example of the successful application of this technique is the Bruker JuiceScreener™ for SGF Profiling™ [1,2].

Traditionally, NMR has been perceived as a tool for structure verification, elucidation and purity analysis. However, driven by the needs of the emerging field of Metabonomics/Metabolomics, NMR has rapidly expanded in recent years into the areas of mixture analysis and screening applications. These developments were facilitated by high-throughput sample changing technology (either sample tube or flow-injection methods), integrated sample preparation, and the improved quality of digital spectrometers in general.

**Figure 1:**
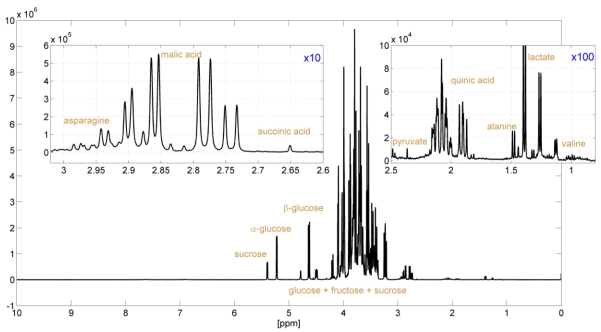
^1^H-NMR spectrum (~4 minutes) of an apple juice and two enlargements (magnification of factor 10 and 100) showing signal assignments of a few key compounds. The x-axis (expressed as ppm relative to 400 MHz) corresponds to the chemical shifts of the proton resonances, which are dependent on the chemical environment of the protons (e.g., type of bonds and neighboring atoms). The y-value is linearly dependent on the molarity of the corresponding proton(s).

Today, NMR is an established tool in a wide range of metabonomics-related applications, including drug toxicity and efficacy screening with animal models [3,4,5], clinical diagnosis of inborn errors [6,7] of metabolism, and general health status screening in the context of epidemiological research [8], to name but a few examples. In such studies, hundreds of samples may have to be screened per day with respect to a) the identity and concentration of selected metabolites and b) for inter-sample comparison of spectral patterns using multivariate statistics to obtain classification and discrimination information. NMR is a particularly well-suited detector for the analysis of biological fluids, providing truly quantitative and structural information while featuring high throughput (a 1D spectrum measured in a few minutes; Figure 1), excellent reproducibility, and minimal sample preparation (typically only addition of buffer).

In this article we demonstrate that the principles established for NMR applications in Metabonomics have been successfully transferred to yet another but closely related field of mixture analysis, *i.e.*, quality control of beverages. A corresponding method for fruit juice analysis, developed in a joint effort by Bruker BioSpin GmbH and SGF International e.V. has been introduced recently under the name SGF Profiling™. The system is fully automated with respect to sample transfer, measurement, data analysis and reporting and is set up on an Avance 400 flow-injection NMR spectrometer. For each juice a multitude of parameters related to quality and authenticity are evaluated simultaneously from a single data set acquired within a few minutes. This multimarker/multi-aspect NMR screening approach features low cost-per-sample and is highly competitive with conventional and targeted juice quality control methods.

Initially, the new NMR-based method was developed as a cost- and time-efficient prescreening tool to identify suspicious samples which may have a quality or authenticity issue and hence require more detailed conventional analysis. However, after having established a spectral database, which currently contains spectra from more than 6,000 reference juices, including about 1,500 fully authentic samples taken by SGF inspectors on site, one can clearly see that the potential of NMR analysis goes far beyond our original intentions.

## 2. Results and Discussion

Evaluation is done in a twofold mode, namely targeted and non-targeted analysis. Targeted in this case means the identification and quantification of individual compounds, whereas the non-targeted analysis approaches apply statistical methods to detect unexpected deviations or to determine the origin of a sample, the fruit content or the addition of other types of fruit. 

### 2.1. Quantification (Targeted Analysis)

In comparison with reference standards, specific deviations in the concentration of a particular compound or in the profile of a specific combination of compounds may indicate characteristic quality and authenticity problems, such as the addition of sugar. Therefore, the primary interest for the food chemist in the classical fruit juice assessment procedure is the concentrations of various ingredients. The targeted approach provides the identification and quantification of individual compounds. Specific deviations in the concentration of a particular compound or in the profile of a specific combination of compounds may indicate characteristic quality problems. Here, NMR spectroscopy provides a clear advantage over classical analysis techniques as it allows the identification and quantification of many compounds in a mixture simultaneously (Figure 1). Our quantification routine provides absolute concentrations for more than 28 different compounds depending on the type of juice (Table 1) *i.e.*, sucrose, glucose, fructose, proline, alanine, 5-hydroxymethylfurfural (HMF), ethanol, methanol, acetone, phlorin, acetaldehyde, benzaldehyde, acetoine, arbutine and the acids malic, citric, isocitric, chlorogenic, lactic, fumaric, quinic, succinic, citramalic, formic, benzoic, acetic, sorbic, gluconic and galacturonic. Furthermore, various useful relationships between compound concentrations are calculated, e.g. the ratio glucose/fructose or the ratio of sucrose to total sugars.

This large amount of analytical results from one measurement readily enables the detection of frauds like the addition of sugar, exhaustive enzymatic treatment (marker: galacturonic acid), addition of citric acid or lemon juice (e.g., in apple juice), extraction of orange peel (marker: phlorin) or the usage of unripe fruits (e.g., high concentration of quinic acid in apple juice).

**Table 1:** SGF Profiling quantification results of an orange juice. The colored flags show agreements with or deviations from reference intervals, which are provided by the European Fruit Juice Association A.I.J.N. (N/Q: Not quantified due to non-detectable signal or insufficient signal assignment).

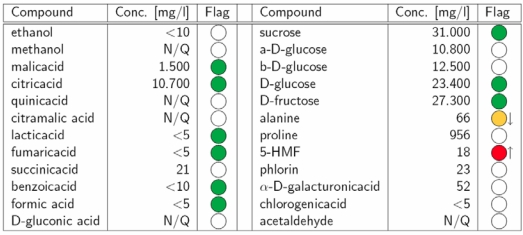

### 2.2. Statistical Analysis (Non-Targeted)

In addition to compound quantification, an exhaustive statistical analysis is applied to the same data, which is based on a large reference database of more than 6,000 samples of more than 50 different types of fruit juices from more than 50 countries. This reference database is constantly growing. A cascade of statistical tests and classification and discrimination steps is applied to scan for multiple aspects of sample quality. 

As a first step, the type of fruit is estimated. The global model can differentiate between 20 types of fruit, namely apple, orange/mandarin, sour cherry, pineapple, black currant, passion fruit, lemon, grapefruit, banana, peach, raspberry, strawberry, pear, apricot, mango, guava, carrot, elderberry, pomegranate and grape. Of course, this information is usually provided with the sample’s metadata and is rarely a reason for complaints, except when orange juice (*Citrus sinensis*) is mixed with mandarin juice (*Citrus reticulata*). The latter is often cheaper so that some companies add it to orange juice without declaration (which is not allowed in Europe). With conventional analysis the addition of mandarin juice to orange juice is difficult to detect, but with our NMR methods and models we can detect mandarin juice at a level of 10% or more and therefore allows a screening prior to DNA- or other conventional analyses.

**Figure 2:**
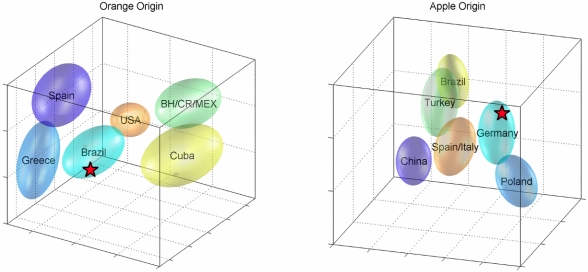
Statistical classification models for the determination of the country of origin. The left plot shows the model for orange juices and the right for apple juices (3D-projections of higher-dimensional spaces). Ellipsoids show confidence spheres for reference subgroups. Star represents actual sample.

With regard to orange juice as well as apple juice *etc.* (see below), more specialized models can distinguish between direct juice and juice from concentrate and can detect the origin of the fruit. Figure 2 (left panel) shows the results for the estimation of origin for a particular orange juice sample. The possible sources or groups included in the model are Spain, Greece, Brazil, Belize/Mexico/Costa Rica, Cuba and USA - other important origins like Argentina or South Africa will be added when more reference samples are available. A 3D projection of the discrimination model space shows the ellipsoids of probability for each source, and the sample of interest represented by a red star. Similarity factors are calculated in the complete discrimination space and provide the probabilities for the estimation of source (the juice is most likely from Brazil). Up to now, we have developed detailed classification models for orange juice, as shown, apple juice (origin; concentrate vs. direct juice), sour cherry and pineapple. The underlying statistical method is a combination of PCA (principal components analysis) and discrimination analysis [10]. The accuracy is checked via cross-validation and Monte Carlo analyses [11].

**Figure 3:**
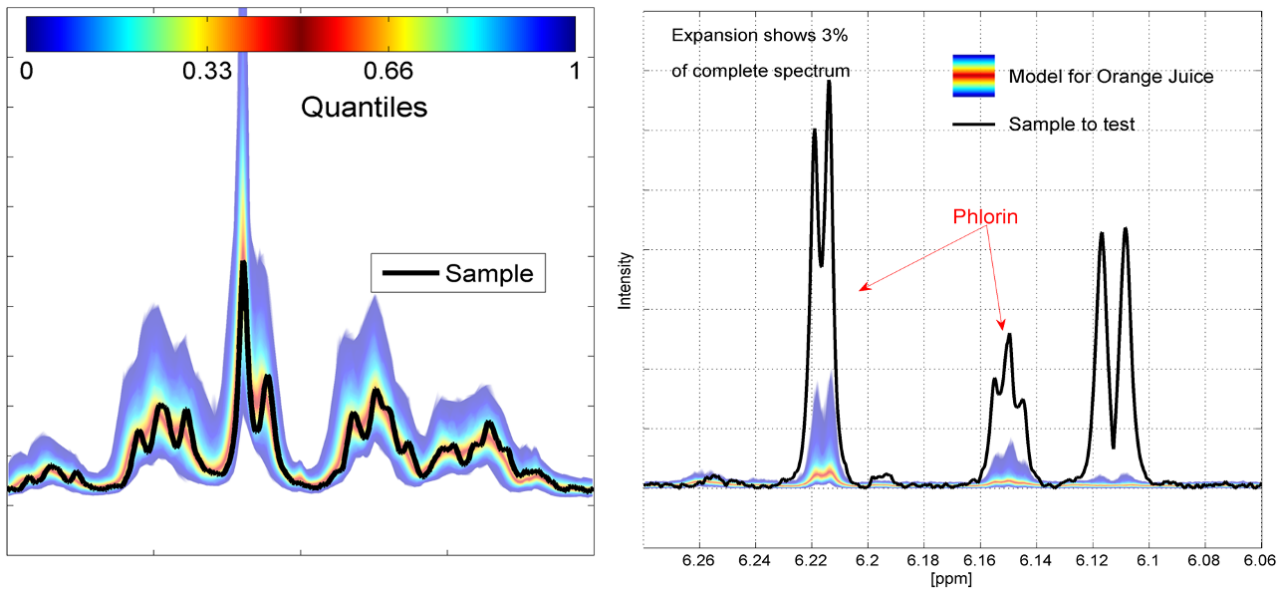
Left panel: Non-targeted verification of the sample (black line) versus the quantile-plot of the respective reference database: apple juice (~1% of spectrum), Right panel: orange juice with high phlorin concentration (indicating over-extraction of the whole orange including its peel).

After the determination of the most likely group assignment, the sample is verified in two steps. First, a univariate analysis compares each spectral region of interest with the reference data set and detects deviations in compound concentrations. Figure 3 (left panel) shows a spectrum expansion around 2 ppm for a rediluted apple juice from China overlaid on a quantiles plot of the reference spectra; any unusual component concentrations can be easily detected (non-targeted analysis, Figure 3, right panel). The second approach is a multivariate analysis based on the PCA/SIMCA approach [10] for detecting deviations which are not apparent in a univariate analysis. If both methods give the same inconspicuous result, the sample is consistent with the models and therefore considered “representative” and has successfully passed the prescreening trial. In this case there is no need to examine the sample further. However, if deviations from normality are detected further analysis also with conventional techniques will be triggered. This non-targeted approach even allows the detection of previously unknown contaminations. 

Another important method for verification, in particular for market samples, is the estimation of the fruit content of the juice. Conventionally, this is done by quantifying selected compounds and minerals and comparing these amounts with reference distributions. In NMR screening, hundreds of variables can be measured on the basis of just one spectrum; hence, we can use regression analysis to estimate the fruit content. Our results have shown that such an analysis yields results with a relative accuracy of about 10% for more than 95% of the samples.

## 3. Experimental Section

Each sample needs minimal preparation effort: addition of buffer (90% juice with 10% buffer, including D_2_O for locking as well as sodium azide to suppress microorganism activity), and in some cases (e.g., for orange juices) centrifugation before that. SGF Profiling is based on an Avance 400 NMR spectrometer with a 9.4-T Ultrashield™ Plus magnet and utilizes flow-injection NMR (BEST™ NMR) with a 4-mm flow-cell probe with Z-gradient and a Gilson liquids handler for sample storage, preparation and transfer. Samples are provided in bar-coded cryovials or well plates, and Gilson cooling racks keep the samples at low temperature (about 4 °C) prior to injection. A heated sample transfer line from the Gilson unit to the probe and a heated capillary inside the probe pre-equilibrate the sample to the desired temperature (e.g., 300 K) during the transfer. Therefore, no additional temperature equilibration time within the measurement cell is needed. 

The overall experimental procedure is controlled by Bruker’s SampleTrack™ software and consists of the following steps, executed under full automation: temperature adjustment, tuning and matching, locking, shimming and the optimization of the pulses and presaturation power for each individual sample. Two NMR experiments are executed: a modified version of the 1D-NOESY sequence that allows quantitative evaluation even close to the water signal (Figure 1) and a fast 2D J-resolved spectrum [12] which facilitates unambiguous signal identification. With modern Bruker NMR instruments baseline correction is redundant and other processing steps such as phase correction and referencing are done in full automation resulting in excellent spectra quality and reproducibility. The whole procedure takes about 15 minutes per sample including sample transfer, measurement, evaluation and reporting.

## 4. Conclusions

In this article we have introduced the SGF Profiling™ method for the authentication, verification and quality control of fruit juices. In addition to the quantification of a large array of characteristic compounds, this fully automated NMR screening technique uses statistical models for the estimation of fruit content or the origin of the juice. This analysis tool can indicate and identify known and unknown deviations from normality.

The reference juice database is updated regularly, so that additional or improved statistical models will be available in the near future for fruit type, fruit origin or other discriminatory factors. Thus, the predictive power of the SGF Profiling™ method will even further be refined and increased over time.

The examples presented here for the screening of fruit juices can also be seen as proof-of-principle for other upcoming applications. The same workflow (preparation, measurement, processing, reporting) and underlying mathematical methods can be easily transferred to other quality control applications, such as the screening of milk, wine or beer, and any other soluble or semi-soluble material [9] as well as to a wide range of clinical/medical applications. Furthermore, all future Metabonomics screening techniques, including clinical diagnosis using biofluids (e.g., urine, plasma, CSF, bile) will benefit from the experience obtained with these fruit juice applications.
